# A Method for Determining Skeletal Lengths from DXA Images

**DOI:** 10.1186/1471-2474-8-113

**Published:** 2007-11-16

**Authors:** Usha Chinappen-Horsley, Glen M Blake, Ignac Fogelman, Tim D Spector

**Affiliations:** 1 King's College London, St Thomas' Hospital Campus, Twin Research & Genetic Epidemiology Unit, Lambeth Palace Road, London, SE1 7EH, UK; 2 King's College London School of Medicine, Guy's Hospital, St Thomas Street, London, SE1 9RT, UK

## Abstract

**Background:**

Skeletal ratios and bone lengths are widely used in anthropology and forensic pathology and hip axis length is a useful predictor of fracture. The aim of this study was to show that skeletal ratios, such as length of femur to height, could be accurately measured from a DXA (dual energy X-ray absorptiometry) image.

**Methods:**

90 normal Caucasian females, 18–80 years old, with whole body DXA data were used as subjects. Two methods, linear pixel count (LPC) and reticule and ruler (RET) were used to measure skeletal sizes on DXA images and compared with real clinical measures from 20 subjects and 20 x-rays of the femur and tibia taken in 2003.

**Results:**

Although both methods were highly correlated, the LPC inter- and intra-observer error was lower at 1.6% compared to that of RET at 2.3%. Both methods correlated positively with real clinical measures, with LPC having a marginally stronger correlation coefficient (r^2 ^= 0.94; r^2 ^= 0.84; average r^2 ^= 0.89) than RET (r^2 ^= 0.86; r^2 ^= 0.84; average r^2 ^= 0.85) with X-rays and real measures respectively. Also, the time taken to use LPC was half that of RET at 5 minutes per scan.

**Conclusion:**

Skeletal ratios can be accurately and precisely measured from DXA total body scan images. The LPC method is easy to use and relatively rapid. This new phenotype will be useful for osteoporosis research for individuals or large-scale epidemiological or genetic studies.

## Background

In modern medicine the use of skeletal ratios has focused mainly on forensic and anthropological studies. The measurement of bone size has a common use in determining age, cause of death, stature estimation and other bodily characteristics in both humans and animals [1]. On a wider scale such data is critical in ergonomics, the bone geometry of the 'average person' being important in the design of human environments and equipment [2]. Although such relationships are important in anthropology and forensic pathology their use in disease prediction has been overlooked despite the evidence they may give about the origins of certain bone-modifying diseases. These may include rickets, osteoarthritis or osteoporosis [3].

The bowing of long bones, characteristic of rickets, for example, results in a reduction in standing height [4]. In osteoarthritis, the growth of new bone alongside old bone also alters shape and length [5]. Moreover, absolute bone lengths vary according to differing body sizes so measurement of a bone ratio would be a better measure for the relationship between bone sites for varying body shapes.

For well over a decade, DXA (dual-energy x-ray absorptiometry) of the spine and hip has been considered the standard test in the diagnosis of osteoporosis [6] where fragility fractures were not evident. It is a quick and cost-effective way of determining fracture risk with little radiation exposure. Bone size has been shown to be an independent risk factor for fracture at a number of sites [7-9] and studies, including those by the St Thomas' Twin Research Unit, have shown that hip axis length measured by DXA may also predict fracture risk independent of bone mineral density (BMD) [10-12]. Furthermore hip axis length has been found to be a heritable risk factor and therefore a useful phenotype for gene discovery [11,13]. In fact, there is already evidence of an association between femoral length and polymorphisms in the RUNX2 gene [14].

A limitation of DXA scans is that they are based on two-dimensional projection images that measure BMD as the mass of bone per unit area. For this reason they do not separate the effects of true bone density (i.e. grams of bone per unit volume) from those of bone size [15]. Nevertheless, DXA scans clearly do contain unused information on bone size (i.e. length and width) that could potentially be used to improve the prediction of fracture risk over and above that given by BMD alone.

Bone length and bone proportions may provide useful additional information since fractures also depend on bone strength and quality and not simply low bone mass [16]. Total body DXA scans have been beneficial in determining fat distribution and muscle mass, for example, in the monitoring of HIV patients during treatment [17]. Some studies have linked stature and skeletal disproportion to cardiovascular disease and diabetes [18]. However, no previous studies have used total body scans to obtain information on skeletal ratios.

On Hologic DXA systems specific areas of a total body DXA scan can be analysed using the special analysis tool that allows the user to create up to seven polygonal regions of interest (ROIs) on the image. Previous studies have used this tool to obtain central body fat composition in the trunk [19]. Other tools used to measure areas on the paper image printout generated from DXA scans are the reticule, a magnifying glass ball which can be placed over the image and used to measure image lengths up to 4 centimetres [20] and a standard 30 cm ruler. Despite the popular use of the special analysis tool in Gruen zone measurements in orthopaedics, particularly in post-operative prosthetic hip studies [21], it has not yet been used to make linear measurements of user-defined ROIs.

The aim of this study was to evaluate the use of both the special analysis tool and the reticule method to determine skeletal lengths for their possible use in epidemiological and genetic studies.

## Methods

### Subjects

Fifty subjects had two DXA whole body measurements taken four years apart. These data were used to test intra-observer error and long-term precision using both linear pixel count (LPC) & reticule and ruler (RET) methods at the same bone sites. In another 20 subjects the validity of the LPC and RET methods was tested by comparing the measurements made on DXA scans with direct anthropometry. In a further group of 20 subjects the LPC and RET methods were compared with measurements made on leg X-rays.

### Reproducibility

Fifty normal subjects with whole body DXA scans (scanned in 1999 and 2003), as part of the TwinsUK Registry at St Thomas Hospital [22-24], were used to test the reproducibility of the two measurement techniques. This population were unselected unrelated female Caucasians between the ages of 18 and 80 years old. Both sets of scans were performed on the same QDR 4500W system (Hologic Inc, Bedford, MA). This scanner's fan-beam geometry could affect width measurements (at right angles to the long axis of the scanning table) despite vertical measures (parallel to the long axis of the scanning table) remaining accurate. Scans were measured for standing height and regional lengths (spine, femur, tibia and radius) using a 4 cm reticule and 30 cm ruler. The same scans were then measured at the same skeletal sites using the special analysis tool on the DXA machine. This is available in the analysis mode where there is an option for sub-regional analysis. Here, the technician can place up to seven polygonal ROIs on the image to isolate various bone and soft tissue areas. Both measurement techniques were applied to scans from 2003 by one observer and repeated a week later to determine intra-observer error. Long-term precision was tested between 1999 and 2003 scans. This error also accounts for the longitudinal change occurring naturally in bone over time. The time taken to use each method was also recorded.

**Figure 1:**
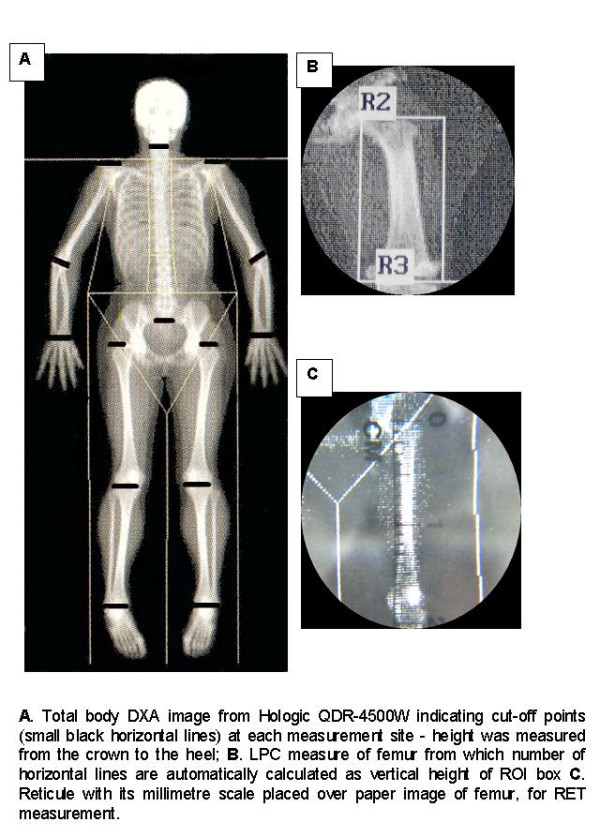
**DXA Imaging: Comparing LPC & RET**. A) Total body DXA image from Hologic QDR-4500W indicating cut-off points (small black horizontal lines) at each measurement site – height was measured from the crown to the heel; B) LPC measure of femur from which number of horizontal lines are automatically calculated as vertical heright of ROI; C) Reticule with its millimetre scale placed over paper image of femur, for RET measurement.

The RET (reticule and ruler) method involved placing the ruler over the consistently sized paper image to measure standing height, from crown to heel, and then positioning the reticule carefully over regional lengths to measure bone sizes. LPC (linear pixel count) was the number of vertical pixels giving the length of the bone. The ROI was a rectangular box for all sites and was adjusted according to the size of the bone (Figure 1).

### Validity

Twenty unrelated Caucasian subjects (18 female; 2 male) visiting the unit during April 2005 were measured using a stadiometer and measuring tape. Measures in centimetres were taken of standing height, sitting height, femur, tibia and radius. The sitting height was taken as a surrogate for the length of the spinal column as it was easier to perform during a twin visit. Bony landmarks in the limbs whilst the subject was in a sitting position were used to measure the femur (greater trochanter to lateral condyle), tibia (lateral condyle to lateral malleolus) and radius (olecranon process of ulna to ulna styloid process).

Leg x-rays obtained in the vertically upright position in 2003 were reviewed for a separate set of twenty subjects. The magnification error was estimated to obtain accurate results. Measurements were taken of the femur and tibia using a 50 cm ruler and an x-ray light box.

To calculate the magnification error in the x-rays, the focus film distance (FFD), object film distance (OFD) and the size of the visual image (VI) were measured. X-ray films were taken of an aluminium step wedge phantom of thickness 40 mm and actual height of 380 mm using a constant FFD of 3000 mm (3 m).

### Statistical methods

The regions explored on the total body scans were standing height, spine, femur, tibia and radius.

Coefficient of variation (CV) was calculated using Excel. The CV was used since it shows differences between methods independent of the units of measurement. Variance comparison tests, calculated using STATA, gave a p-value to indicate the significance of the differences between methods when bone lengths were re-scaled to a mean of 1. The analysis tested the null hypothesis that both methods had the same CV.

Since it was necessary to perform multiple testing and the variables being analysed were highly correlated, the number of effectively independent tests performed for repeated analyses of correlated variables was calculated by utilising a simple linear regression adjusted r^2 ^statistic [25].

## Results

Collectively, the subjects were scanned between 1999 and 2005. Base-line physical descriptors are shown in Table 1. The time taken to use LPC during each test was half that of RET at 5 minutes per scan.

**Table 1:** Baseline Physical Descriptors

Demographics **(*n *= 50)**	**Baseline Mean ± SD**
**Age (years)**	57.8 ± 8.42
**Height (cm)**	1.61 ± 0.06
**Weight (kg)**	69.5 ± 12.01
**BMI (kg m^-2^)**	26.6 ± 4.50

Nominal p-values were evaluated for 19 statistical tests: variance ratio tests for standing height, spine, femur, tibia and radius (for both intra-observer error and long-term precision) and standing height, sitting height, femur, tibia and radius (for real measures) and left and right femur and tibia (for X-rays). However, rather than applying the Bonferroni correction which would be over-conservative since the tests are related, the effective number of tests was calculated using simple linear regression [25]. Multiple testing showed that although 19 statistical tests were performed in total this was equivalent to 6.3 independent tests. The Bonferroni correction was then applied to calculate the p-value: α = 0.05/6.3 = 0.008.

In deducing magnification error for the leg X-rays OFD was measured to the centre of the phantom. In contact, with an OFD of 20 mm, the VI was 380 mm. Simulating an average examination, at an OFD of 100 mm, the VI was 395 mm giving a magnification error of 3.9%. In the 'worst-case scenario', with an OFD of 200 mm, the VI was 410 mm giving a magnification error of 7.9%.

### Reproducibility

**Figure 2:**
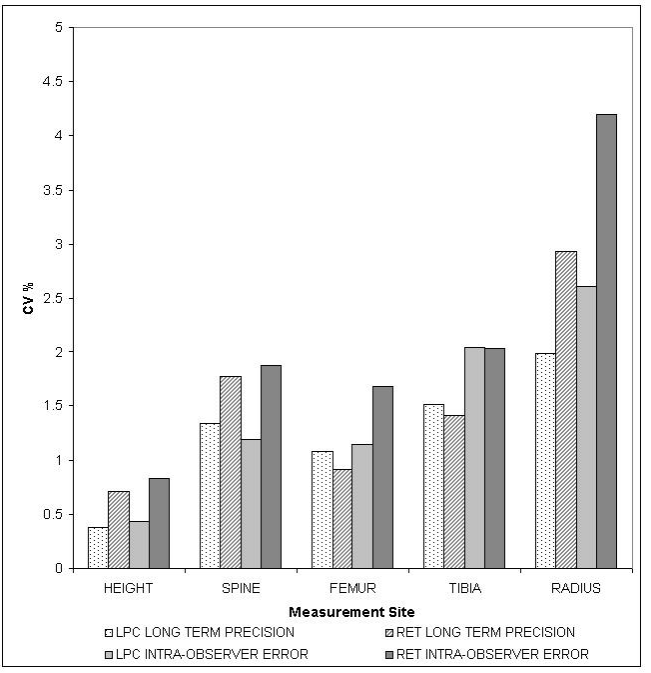
A bar graph of the coefficient of variation of bone lengths (%) against measurement sites for LPC and RET methods. Long-term precision was measured between scans performed in 1999 and 2003 and intra-observer error was personal reproducibility measured after one week using scans performed in 2003.

**Figure 3:**
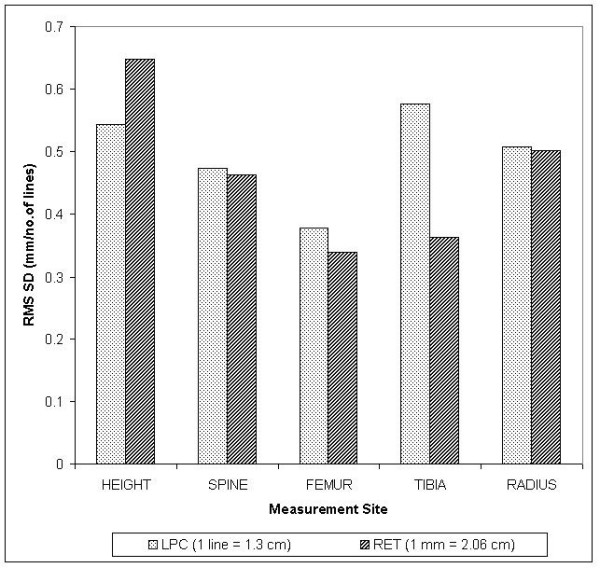
A bar graph of RMS SD against region of measurement for LPC (no. of lines) and RET (mm) methods.

Fifty females were used to test observer error. When the coefficient of variation (CV) was calculated for long-term precision and intra-observer error using both methods (Figure 2), mean CV% for LPC (1.6%) was lower than RET (2.3%). Although units of measurement were different in each method, comparison of these true measures using the root mean square standard deviation (RMS SD) shown in Figure 3 indicates that deviation for the LPC and RET were similar for the same sites, except for the tibia where the LPC was higher than all other measures. For RET, 1 mm was equivalent to approximately 2.06 cm and for LPC, 1 line was equivalent to approximately 1.3 cm.

The null hypothesis that the LPC and RET variances were equal was tested using the variance ratio test. Applying the Bonferroni correction (p-value = 0.008), the five regions tested (standing height, spine, femur, tibia and radius) showed no significant difference between the CVs for the two methods.

**Figure 4:**
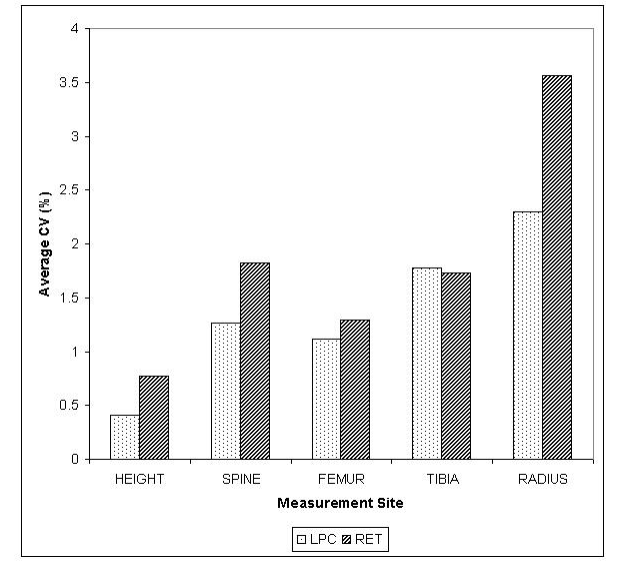
A bar graph showing average CV% (long-term precision and intra-observer error) between five bone measurement sites using LPC and RET. The largest average CV% belongs to the radius.

Combined CV% was the overall average CV% between long-term precision and intra-observer error at each site. At individual measurement sites (Figure 4) the lowest average CV% was the LPC measurement of standing height (0.4%). The highest average CV% was 3.6% for the RET measurement at the radius.

### Validation

**Figure 5:**
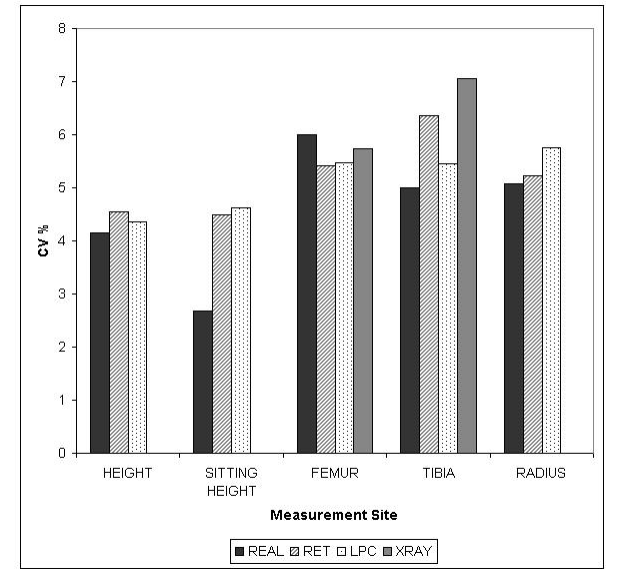
A bar graph showing combined population CV% between five bone measurement sites using four different measurement techniques – Real, RET, LPC and X-ray (only performed on femur and tibia). Real measures have the lowest CV% at four out of five sites.

Twenty real measures and twenty x-rays were compared to LPC and RET. Coefficient of variation between methods showed, as expected, real measures to have the lowest CV% (4.6%) and x-ray measures to have the highest CV% (6.4%). LPC and RET had an average CV% of 5.1% and 5.2% respectively (Figure 5).

The null hypothesis that the population CV of the two methods were equal was tested using a variance ratio test. The five regions tested (standing height, sitting height, femur, tibia and radius) agreed with the null hypothesis and showed there was no significant difference in CVs using the Bonferroni correction (p-value = 0.008).

Regression analysis resulted in high positive correlation coefficients for both methods compared to real and x-ray measures. LPC performed slightly better (r^2 ^= 0.94; r^2 ^= 0.84; average r^2 ^= 0.89) than RET (r^2 ^= 0.86; r^2 ^= 0.84; average r^2 ^= 0.85) with x-rays and real measures respectively. These differences were not statistically significant.

## Discussion

Both methods tested correlated with each other and were found to be good and reproducible methods of measuring skeletal sizes. Nevertheless, LPC generally performed better with long-term precision and less intra-observer error than RET and with stronger correlation coefficients when compared to real measures. Moreover, the LPC method saved time in both the extraction and analysis of DXA images at 5 minutes per scan which was half the time of the RET measure.

Although RET is a method easy to use anywhere because it is so simple – the paper image, one ruler and one reticule – it is a less precise method of measurement. In addition, the paper images are not as clear as those on screen and cannot be manipulated to darken or lighten the image contrast whilst retaining resolution, as in the LPC method. Nevertheless, observations of RMS SD (Figure 3), display the same pattern of deviation (excluding standing height) for both methods. Consequently, RET is a good substitute for LPC.

There are several potential limitations in this study. The reproducibility results only apply to Caucasian women. Gender, ethnic and socio-economic differences that may not be visible here could account for varying results in ratio or length [26-29].

Long-term precision and intra-observer error exists between operators over time [30]. Variation in patient positioning may lead to random differences between images and may be due to many reasons including operator training or arthritic pain. BMI can also have an effect on the average standing height of the subject above the scanning table and this can result in a variation in image dimension across the table in fan beam systems.

Despite variations in the distance from the x-ray source to the film having only a minor effect on magnification, small changes in leg thickness may increase magnification error. In a 'worst-case scenario', where the OFD is increased to a distance equivalent to that of a subject with an extremely high BMI, the results show the error of magnification to be approximately 8%. In an average x-ray, where the OFD with subjects having an approximately normal BMI is less, the results show the error of magnification to be 4%. Since the BMI of these subjects was just over average at 26.6 kg/m^2^, it was assumed that the error of magnification at 4% would be inconsequential to x-ray accuracy for the purposes of this study.

Furthermore, there was greater variation in the spine than in any other skeletal site. This may be attributable to bone and joint diseases and caution must be taken in those aged over 65 years as the spine may be affected by osteoarthritis or fractures. Moreover, any scoliosis, kyphosis or lordosis would cause standing height differences where there is bending or crushing in the vertebral column. Obvious differences were accounted for in this subject group.

One of the major problems encountered in measurement of total body DXA images is the positioning of the forearm. The forearm should be positioned in parallel to the long axis of the scanner table (Figure 1) yet, in some cases, was not. This is normally due to the larger patient's body size resulting in the forearm being positioned at an angle. The measurements were corrected using Pythagorus' Theorem. Using the special analysis tool a triangle was constructed from the upper tip of the olecranon process to the lower tip of the ulna styloid process. Image distortion that occurs with Hologic fan-beam bone densitometers will affect horizontal but not vertical measurements. Bone width was not measured in the present study but will be evaluated in future research by dividing LPC measured bone area by bone length.

## Conclusion

In conclusion, we have shown that it is possible to measure bone length reliably from DXA studies. The LPC is a faster and more consistent method to use when measuring skeletal size from total body DXA scans. This should encourage its wider use in clinical research. Future studies may be able to use this novel LPC method to create large databases of bone ratios, geometry and anthropometry from existing scans. These databases should lead to a wide number of studies particularly in the field of genetics and epidemiology of osteoporosis.

## Competing interests

The author(s) declare that they have no competing interests.

## Authors' contributions

UCH was involved with the original concept, planning the study, data extraction, analysis and preparing a manuscript. GMB, IF and TDS were involved with the original concept, planning and advice. All authors read and approved the final manuscript.

## Pre-publication history

The pre-publication history for this paper can be accessed here:


